# Supplementary material for: Low-Level Tolerance to Antibiotic Trimethoprim in QAC-Adapted Subpopulations of Listeria monocytogenes
Source: Foods. 2021 Aug 4;10(8):1800. doi: 10.3390/foods10081800 (PMC8393223; doi:10.3390/foods10081800)
Supplement: Supplementary file 1 [file foods-10-01800-s001.zip › foods-1277551-supplementary.pdf]

foods MDPI

Supplementary Material

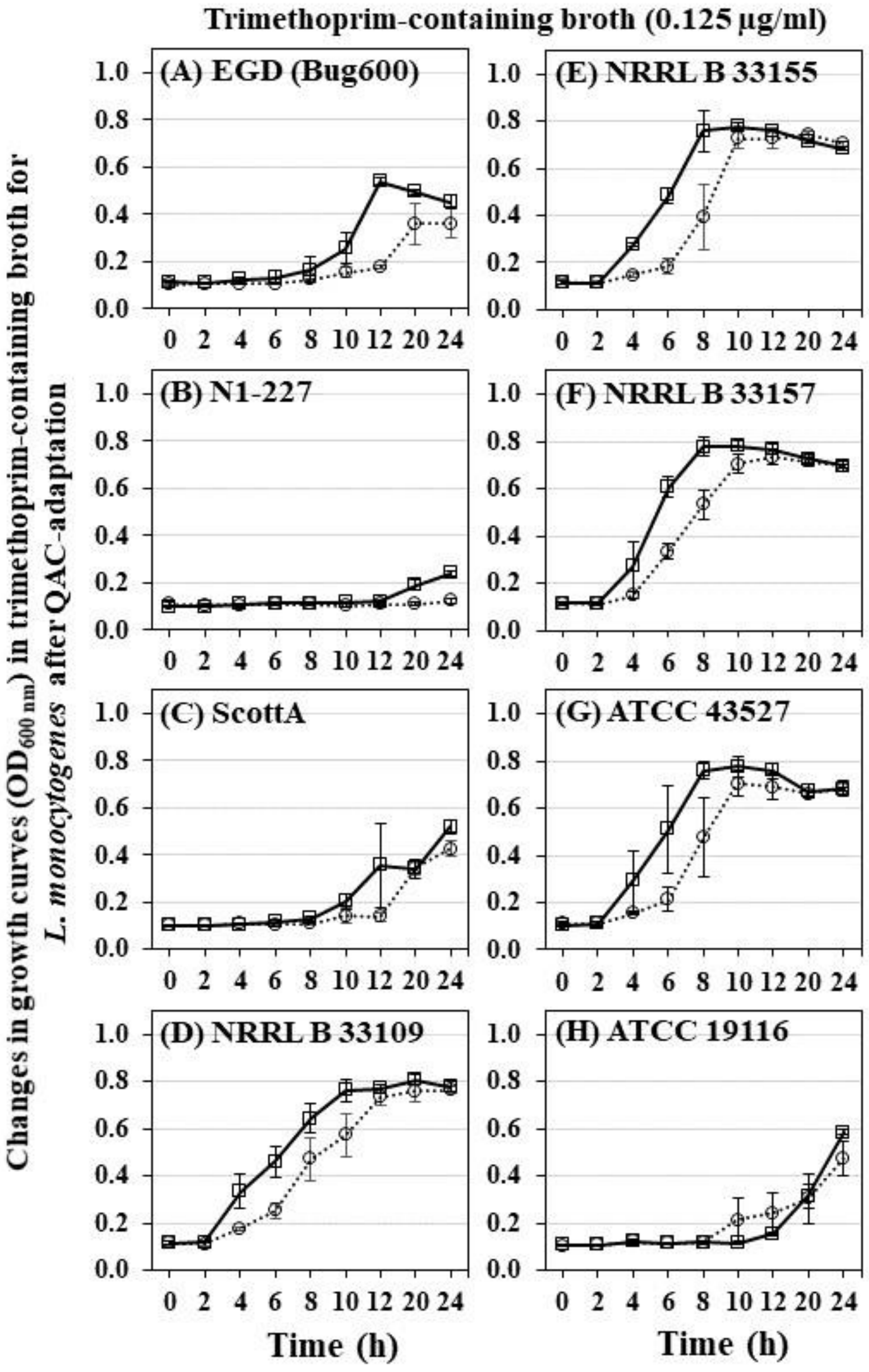

Figure S1. Changes in growth rate (OD_{600}) in trimethoprim (at 0.125 µg/ml in TSBYE) of QAC-adapted subpopulation 1 (open squares) compared to non-adapted control (open circles) cells of eight *L. monocytogenes* strains at 37°C: (**A**) EGD (Bug600), (**B**) N1-227, (**C**) ScottA, (**D**) NRRL B 33109, (**E**) NRRL B 33155, (**F**) NRRL B 33157, (**G**) ATCC 43527, (**H**) ATCC 19116. Error bars indicate standard error of means.

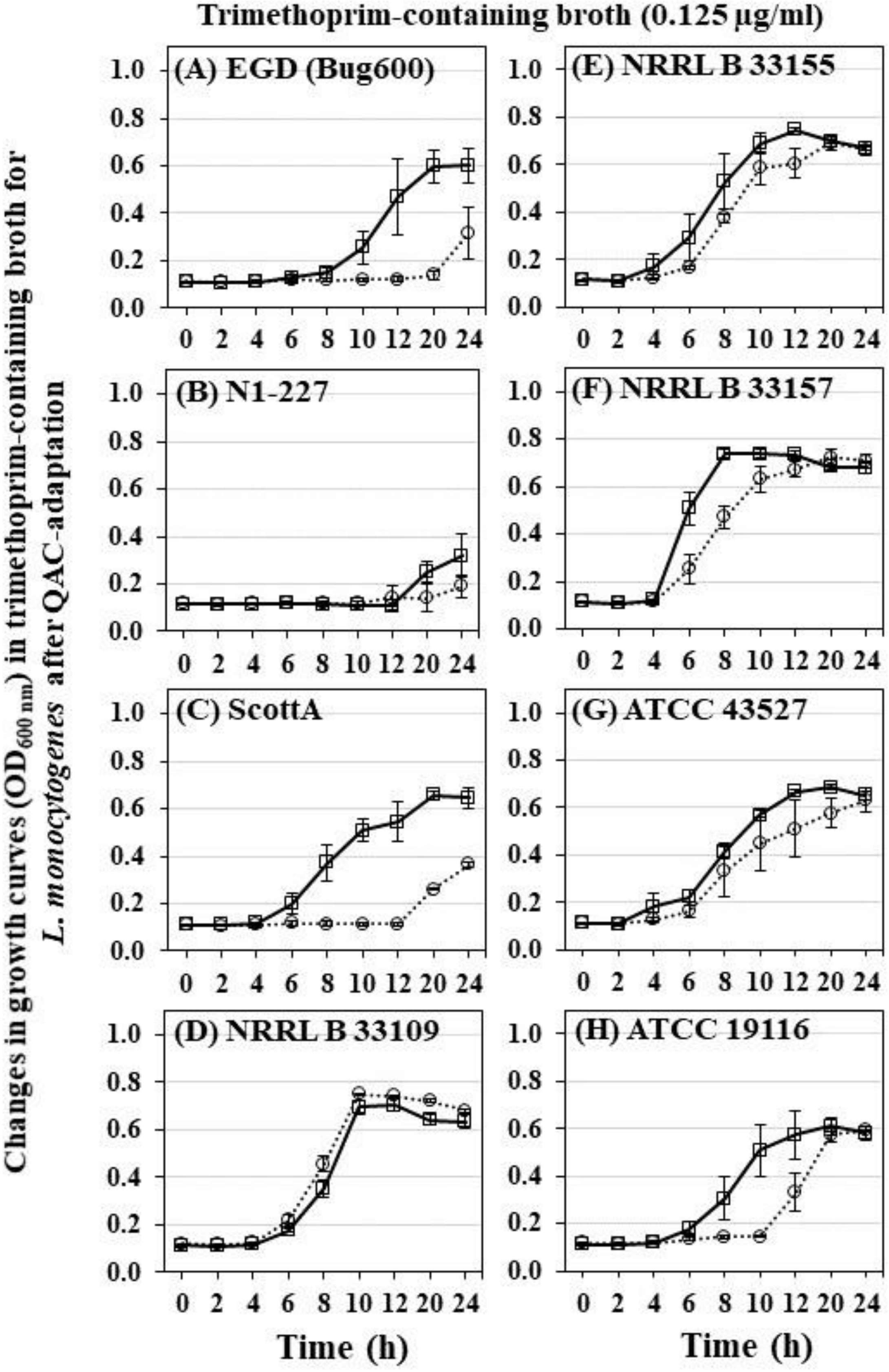

Figure S2. Changes in growth rate (OD_{600}) in trimethoprim (at 0.125 μg/ml in TSBYE) of QAC-adapted subpopulation 2 (open squares) compared to non-adapted control (open circles) cells of eight *L. monocytogenes* strains at 37°C: (**A**) EGD (Bug600), (**B**) N1-227, (**C**) ScottA, (**D**) NRRL B 33109, (**E**) NRRL B 33155, (**F**) NRRL B 33157, (**G**) ATCC 43527, (**H**) ATCC 19116. Error bars indicate standard error of means.

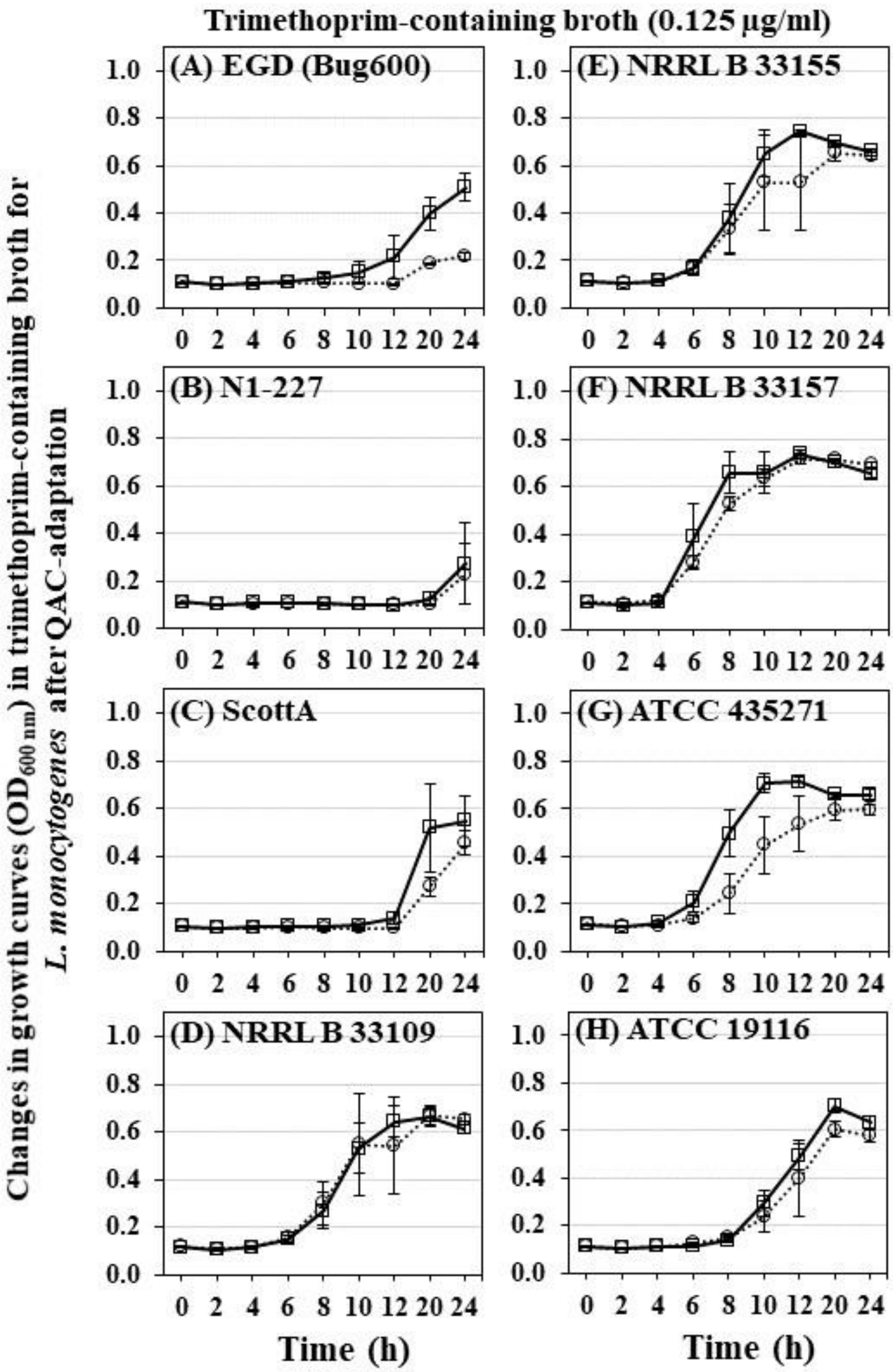

Figure S3. Changes in growth rate (OD600) in trimethoprim (at 0.125 μg/ml in TSBYE) of QAC-adapted subpopulation 3 (open squares) compared to non-adapted control (open circles) cells of eight *L. monocytogenes* strains at 37°C: (**A**) EGD (Bug600), (**B**) N1-227, (**C**) ScottA, (**D**) NRRL B 33109, (**E**) NRRL B 33155, (**F**) NRRL B 33157, (**G**) ATCC 43527, (**H**) ATCC 19116. Error bars indicate standard error of means.
